# Time-Of-Flight Camera, Optical Tracker and Computed Tomography in Pairwise Data Registration

**DOI:** 10.1371/journal.pone.0159493

**Published:** 2016-07-19

**Authors:** Bartlomiej Pycinski, Joanna Czajkowska, Pawel Badura, Jan Juszczyk, Ewa Pietka

**Affiliations:** Faculty of Biomedical Engineering, Silesian University of Technology, Zabrze, Poland; The Lee Kong Chian School of Medicine, SINGAPORE

## Abstract

**Purpose:**

A growing number of medical applications, including minimal invasive surgery, depends on multi-modal or multi-sensors data processing. Fast and accurate 3D scene analysis, comprising data registration, seems to be crucial for the development of computer aided diagnosis and therapy. The advancement of surface tracking system based on optical trackers already plays an important role in surgical procedures planning. However, new modalities, like the time-of-flight (ToF) sensors, widely explored in non-medical fields are powerful and have the potential to become a part of computer aided surgery set-up. Connection of different acquisition systems promises to provide a valuable support for operating room procedures. Therefore, the detailed analysis of the accuracy of such multi-sensors positioning systems is needed.

**Methods:**

We present the system combining pre-operative CT series with intra-operative ToF-sensor and optical tracker point clouds. The methodology contains: optical sensor set-up and the ToF-camera calibration procedures, data pre-processing algorithms, and registration technique. The data pre-processing yields a surface, in case of CT, and point clouds for ToF-sensor and marker-driven optical tracker representation of an object of interest. An applied registration technique is based on Iterative Closest Point algorithm.

**Results:**

The experiments validate the registration of each pair of modalities/sensors involving phantoms of four various human organs in terms of Hausdorff distance and mean absolute distance metrics. The best surface alignment was obtained for CT and optical tracker combination, whereas the worst for experiments involving ToF-camera.

**Conclusion:**

The obtained accuracies encourage to further develop the multi-sensors systems. The presented substantive discussion concerning the system limitations and possible improvements mainly related to the depth information produced by the ToF-sensor is useful for computer aided surgery developers.

## Introduction

Image-guided surgery requires all system components to be aligned and displayed in one coordinate system. The alignment should be performed by the operating room real-time applications, assisting the interventions. They mostly employ pre- and intra-operative imaging modalities. Actions preceding the surgery usually involve scanning the anatomical volume of interest using computed tomography (CT), magnetic resonance imaging (MRI), etc. The raw image data obtained as a result might be used directly for treatment purposes, yet additional processing is usually employed. The intra-operative stage requires real-time acquisition devices and information techniques able to process the data, and to align with one another and with the pre-operative information via registration [1, 2]. Several modalities might be implemented here, *e.g.* ultrasonography (USG) [3, 4], endoscopy [5, 6], bronchoscopy [7], visual navigation systems [8–11], or time-of-flight (ToF) cameras [12, 13]. The issues of equipment synchronization, mutual spatial data correspondence, and finally the registration algorithms are covered by the intra-operative computer-aided surgery (CAS) systems designed for specific purposes [14]. A registration process matches the image data to the patient by finding te rotation and translation matrix between the two physical spaces [1]. Various studies have been conducted to solve this problem.

The first group of the online registration studies applied in commercial systems involves fiducial markers attached to anatomical landmarks [15]. Their location tracked by specific navigation devices is referred to the pre-operative image data [5, 16]. Those systems require, however, a well defined and repeatable landmark specification and placement, and promise the better results, the more rigid the anatomical object of interest is [17]. The common problem of fiducial markers attachment is its physically invasive character, always causing some level of danger during the treatment. In the past years those two limitations have been studied and other propositions have been formulated, mostly for the more demanding soft tissue surgery [18, 19]. The noninvasiveness requirement is being overcome by the surface matching techniques replacing marker matching approaches [20]. The ToF-camera is a device suitable for surface tracking and matching to the preprocessed data [21], and several attempts to employ it in an intra-operative computer aided diagnosis and therapy have been reported recently [22].

The ToF-camera measures the depth of a 3D scene ahead using the infrared light source and CCD detector [21]. The imaging idea uses the multi-detector measurement of the optical signal generated by the device and reflected by the scene. The scene is mostly represented by a cloud of points with their Cartesian coordinates reflecting the distance to the camera. The CCD resolutions have increased from not more than 100 × 100 in original applications [21] to ca. 640 × 480 currently [23]. The depth resolution relies mainly on the source light frequency and distance to the scene and barely reaches 1 cm and less [23]. The ToF measurement still meets substantial challenges. Low image and depth resolution, systematic or intensity-related distance error, depth inhomogeneity, motion artefacts, multiple scene reflections, unseen zones in concave objects and clutter are the main ones [21, 23]. Nonetheless, the ToF-camera measurement speed, interpretation simplicity and noninvasiveness stimulate the intra-operative research in terms of multimodal image guidance.

Registration of the intra- and pre-operative imaging data requires the object of interest to be represented by a surface or a cloud of points in both modalities [24, 25]. Many algorithms have been designed for preoperative processing of medical studies in terms of semi-automatic or automatic segmentation [26–29] or data transformation into some required format (*e.g.* volumetric or surface representation of anatomical structures under consideration [30] or a patient-specific model [31–33]). The matching algorithms attempt to fit the surfaces as tight as possible according to some defined accuracy metrics, *e.g.* Hausdorff distance as well as mean absolute distance, indicating either the largest or mean spatial interval between surfaces [34–36]. Depending on the required level of accuracy, the registration might be treated as rough or fine [13]. However, the registration features an important challenge related to the inability to predict the primary pose correspondence between the optically observed shape and its virtual version prepared on the basis of a pre-operative scan. That is why many applications assume, that a rough registration step has been performed before launching the fine matching algorithm in either way: manually [37], with fiducial markers architecture [38, 39], or via automatic segmentation and landmarks determination with some rigidity constraints [40]. In general, the matching relies on a selection of corresponding feature points in both surfaces [41]. A local neighbourhood of feature points is then represented by descriptor vectors. Based on descriptor similarities, the surfaces are aligned to each other using some predefined similarity metrics, yielding a transformation formula [42]. Due to the intra-operative performance, some surface matching problems appear more noticeable, *e.g.* non-rigidity of structures of interest, distortions, noise or partial visibility leading to a lack of surface and landmarks [13]. Thus, a high level of inconsistency has to be assumed and dealt with during the extraction of feature points. Among the fine registration techniques, the Iterative Closest Point (ICP) algorithm [3, 43–45] seems to be the most widely used. The algorithm is convergent, as it iteratively tracks the point correspondences between the datasets and recalculates the rigid transformation formula in order to minimize the Euclidean distance. Since we use the ICP registration as an important component of our system, we leave its description for Section Data registration.

Operating room registration approaches involving ToF-camera mostly attempt to relate its signal to the pre-operative, pre-segmented CT [13] or MRI [46] data. However, the ToF-assisted medical applications have not left the laboratory tests phase so far. The registration systems employing ToF have been used for matching it with 3D endoscopy image in laboratory set-up [47], or in an intra-modality ToF-to-ToF approach [48]. The latter study describes a rigid registration system for an operating room application. Their framework has been validated using a live dataset acquired by a ToF device and registered with the reference data using a plaster cast body phantom. Generally, the reference dataset has been defined as static pretreatment data in terms of an observed surface, yet in fact it has been another cloud of ToF points, acquired with a different arrangement. The quantitative evaluation relied on the target registration error (TRE), defined as the Euclidean distance between the translational components and absolute translation angle error. To the best of our knowledge no such studies have been conducted so far on the ToF and marker-driven optical navigation correspondence.

This paper presents a novel study on surface matching using CT, ToF and an optical navigation system. A registration procedure presentation is followed by the evaluation of matching accuracies of each of the three pairs of modalities in terms of Hausdorff distance and mean absolute surface distance using phantoms of various human organs. We believe, that connection of those three different acquisition systems promises to provide a valuable support for operating room procedures. The preoperative medical imaging, *e.g.* the CT, plays a big role in treatment planning. The optical navigation system stands for a reliable positioning tool [11]. Finally, the noninvasive ToF depth measurement offers a number of points as a surface representation, matchable to the CT-segmented structures. The obtained results give the system user a feedback and overall view concerning the usefulness of a described set-up. This is also a preliminary study on deploying the ToF-camera as a replacement of the optical tracker’s pointer tool at the object calibration stage.

## Materials and Methods

### Experimental set-up

**Fig 1:**
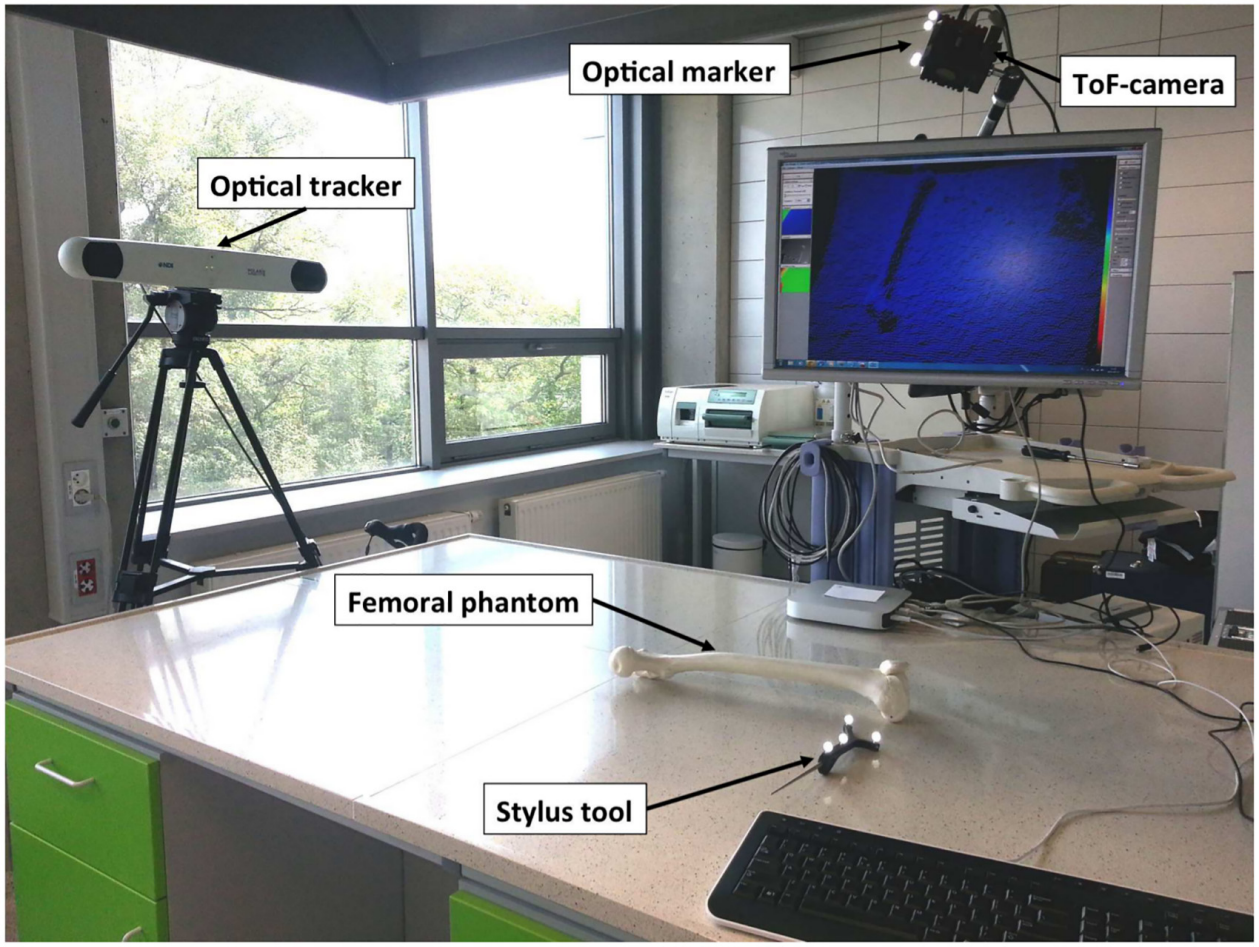
Experimental setup.

The registration system (Fig 1) consists of three different data sources used to represent the object of interest: SwissRanger SR4000 ToF-camera (MESA Imaging AG, Switzerland, http://www.mesa-imaging.ch), the Polaris Spectra navigation system (Northern Digital Inc., ON, Canada, http://ndigital.com) and the CT scanning in a pre-operative mode. These three acquisition techniques are implemented to receive three point clouds of an object. In this study phantoms of the following human organs have been employed: (1) the femur and patella, (2) the upper limb, (3) the head, and (4) the breast. The phantoms are made of various plastic materials, both rigid and flexible.

The ToF-sensor produces a depth data matrix ((*x*, *y*, *z*) coordinates) at 176 × 144 pixel resolution as well as the amplitude (intensity) image and a confidence value for each acquired point. The optical tracker finds the position and orientation of a tool by following the optical marker location. Once sliding the tool along the phantom, its surface is scanned, yielding point cloud.

### Registration system

The registration system employing ToF and marker-driven optical tracker requires a fast, robust and repeatable calibration procedure. Thus, the intrinsic parameters of the ToF-camera as well as the position of the ToF-camera within the tracker coordinate systems (extrinsic parameters) are found.

**ToF-camera intrinsic parameters** The ToF-camera acquires the image depths as well as grayscale intensities of corresponding pixels. With these images, the camera pose is established using OpenCV toolkit (http://opencv.org) according to the pinhole model. Intrinsic parameters are the camera features, which do not depend on the scene viewed, but only on the camera optics itself. They include the focal length, principal point of the optical axis, distortion coefficients. Once estimated, they are valid until the focal length (*i.e.* zoom) changes.

Computation of intrinsic parameters is performed in a standard way with a set of grayscale chessboard images [49] using the implementation provided by OpenCV. Due to the low contrast and spatial resolution of images (144 × 176 pixels), the intensity rescaling as well as the image upsampling for subpixel corners detection are performed.

**ToF-camera extrinsic parameters** Once the intrinsic parameters are given, the absolute orientation of the camera in the external (*i.e.* global) coordinate system can be found. Following the pinhole camera model [49], the relationship between the 3D homogeneous point $P^{G}={\left[x^{G},y^{G},z^{G},1\right]}^{T}$ in a global coordinate system and its 2D image projection [*u*, *v*, 1]^*T*^ is given by:

$$\begin{array}{c} s\left[\begin{array}{c} u\\ v\\ 1 \end{array}\right]=M_{I}M_{E}\left[\begin{array}{c} x^{G}\\ y^{G}\\ z^{G}\\ 1 \end{array}\right], \end{array}$$ (1)

where *M*_*I*_ and *M*_*E*_ are the matrices of intrinsic and extrinsic coefficients, respectively, and *s* is the scale factor. The correction of lens distortions is performed according to [49].

To compute the matrix of extrinsic parameters, one has to match the set of 3D points recorded by the tracker and their corresponding coordinates in the ToF intensity image, known as,,Perspective-n-Point problem” (PnP) [50]. For this, the inner corners of the calibration chessboard are used, since they can be easily detected in the image and their position in the tracker coordinate system can be precisely acquired with pre-calibrated stylus tool.

The extrinsic parameters matrix *M*_*E*_ can be extended to a rigid-body transformation matrix $T_{G}^{C}$ by adding a row [0, 0, 0, 1] at the bottom. $T_{G}^{C}$ is orthogonal and denotes rotation and translation of the camera with respect to the global coordinate system (Fig 1). The coordinates of a point $P^{G}=\left(x^{G},y^{G},z^{G}\right)$ given in global coordinate system can be transformed into a point $P^{C}=\left(x^{C},y^{C},z^{C}\right)$ in the ToF-camera coordinate system C:

$$\begin{array}{c} P^{C}=T_{G}^{C}P^{G}. \end{array}$$ (2)

The direction of transformation can easily be inverted:

$$\begin{array}{c} P^{G}=T_{C}^{G}P^{C}, \end{array}$$ (3)

where $T_{C}^{G}={\left(T_{G}^{C}\right)}^{-1}$.

**Fig 2:**
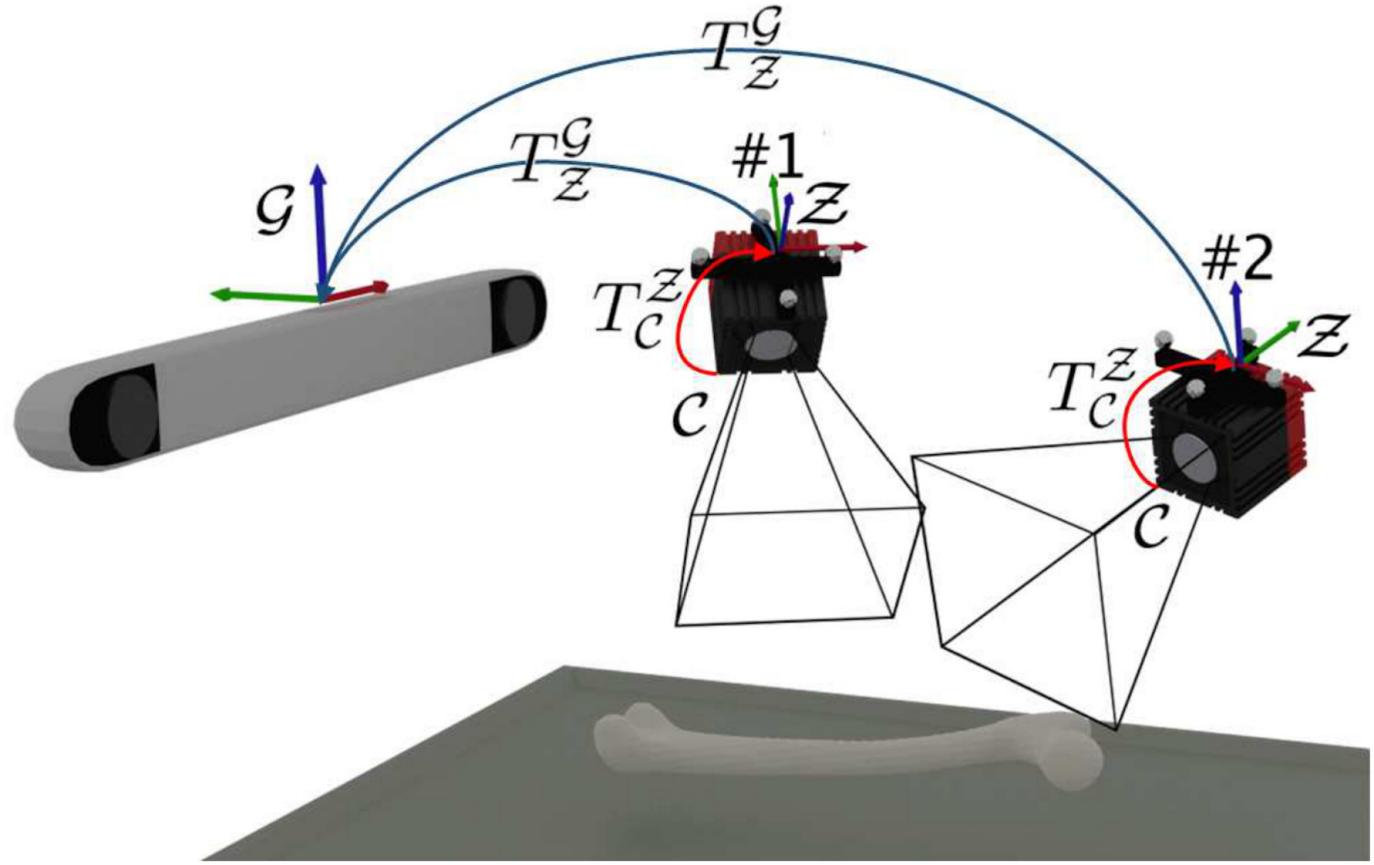
Transformations between different coordinate systems. Once estimated, the transformation $T_{C}^{Z}$ between ToF-camera C and the marker Z coordinate systems remains invariant, despite of the camera movement relative to the global coordinate system G.

Since the extrinsic parameters are related to global coordinate system, their values are valid only as long as the spatial relation between the ToF-camera and the tracker does not change. To make the processing stable and universal, an additional coordinate system is introduced. It remains invariant with respect to the ToF-camera, regardless of the camera movement in a global tracker space. The coordinate system is constructed by the optical tracker marker fixed onto the ToF-camera. This enables the extrinsic parameters determination with respect to the marker and then both, the tracker and the ToF-camera can freely be moved around (Fig 2). Therefore, the extrinsic parameters matrix denotes the transformation $T_{C}^{Z}$ of points registered by the camera (coordinate system C) into the camera marker coordinate system Z:

$$\begin{array}{c} P^{Z}=T_{C}^{Z}P^{C}. \end{array}$$ (4)

As long as the camera marker remains visible for the tracker, the transformation ${\left(T_{Z}^{G}\right)}_{\tau }$ between the marker and global coordinate system in time *τ* is known. Then, we can transfer each point acquired by the ToF-camera directly into the global coordinate system:

$$\begin{array}{c} P^{G}={\left(T_{Z}^{G}\right)}_{\tau }T_{C}^{Z}P^{C}. \end{array}$$ (5)

### Data registration

**Fig 3:**
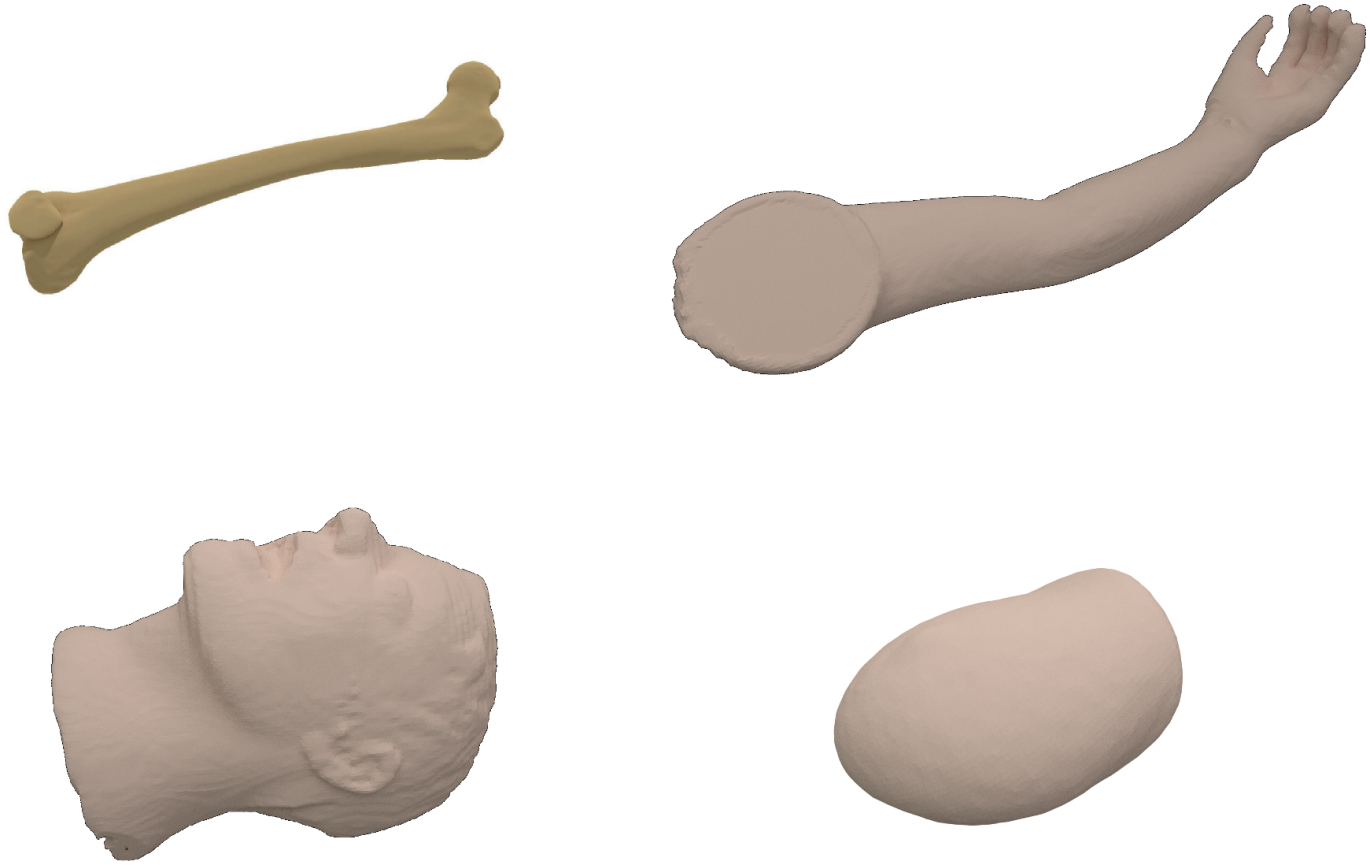
CT surfaces extracted from the 3D segmentation results.

**Data pre-processing** Both, the preoperative CT and ToF data require robust segmentation procedures in order to minimize the influence of noise and unwanted structures within the acquired data. The CT segmentation step uses a thresholding technique based on Hounsfield units supported by mathematical morphology to extract the 3D phantom object. Since all phantoms subjected to the segmentation feature the mean density over 0 HU, they are extracted from the surrounding air (density not exceeding −800 HU) using the automatic Otsu thresholding technique [51] (threshold values ranged between −600 and −550 HU) followed by morphological corrections and 2D/3D connected component analysis. Since the registration step requires a surface object representation, the outer surface is extracted from the object (Fig 3).

**Fig 4:**
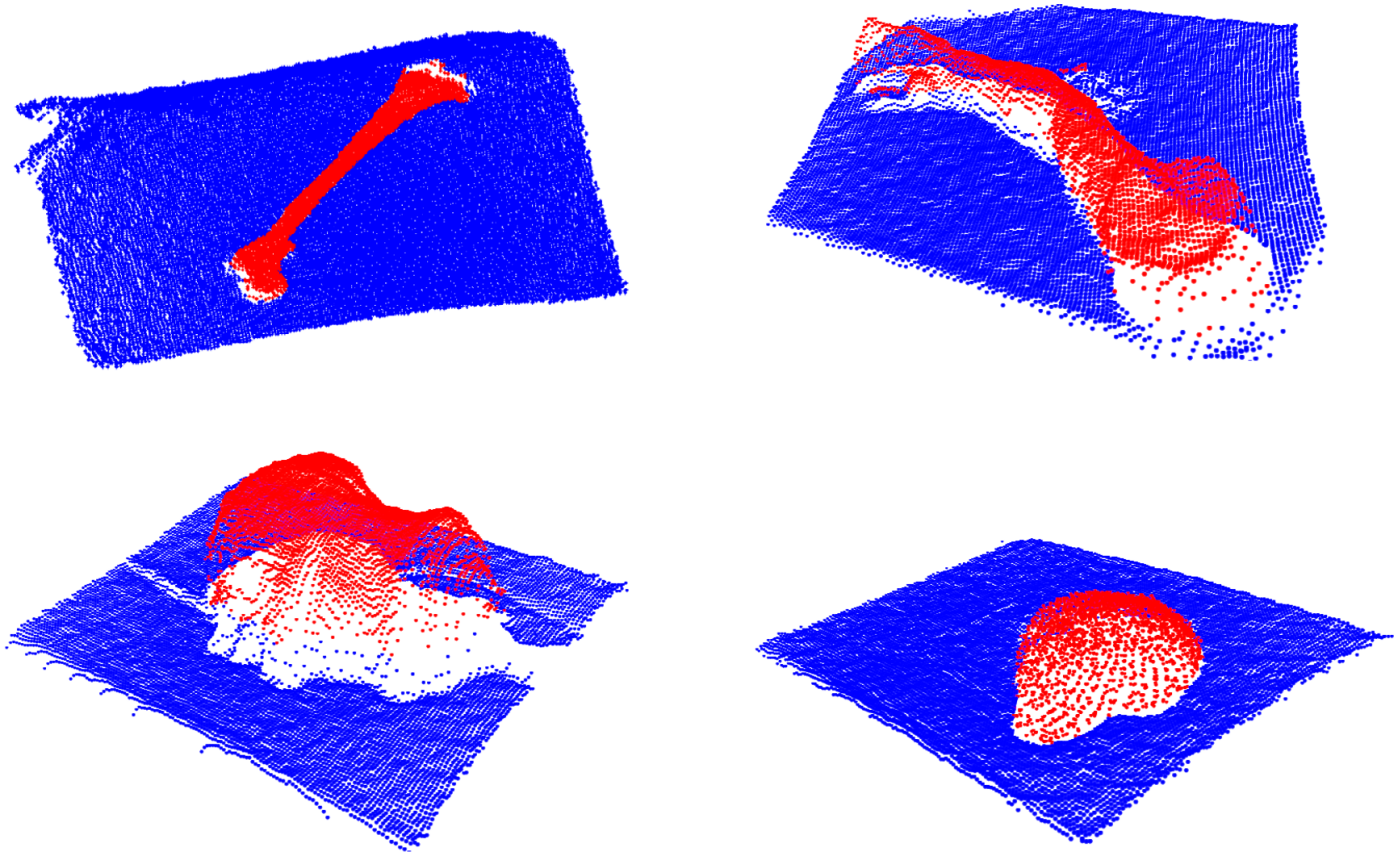
ToF depth images segmentation results in a 3D view.

The other 3D segmentation algorithm is applied to the ToF data analysis. In order to reduce the effect of depth inhomogeneity, which leads to incorrect distance values at object boundaries (“flying pixels”) [21], the confidence map provided by the camera is used. The point coordinates within the ToF-camera coordinate system and amplitude image are merged in the feature space. Such a two-element feature vector is then subjected to a Weighted Fuzzy C-Means (WFCM) [4, 52] clustering procedure leading to segmentation results shown in Fig 4.

**Fig 5:**
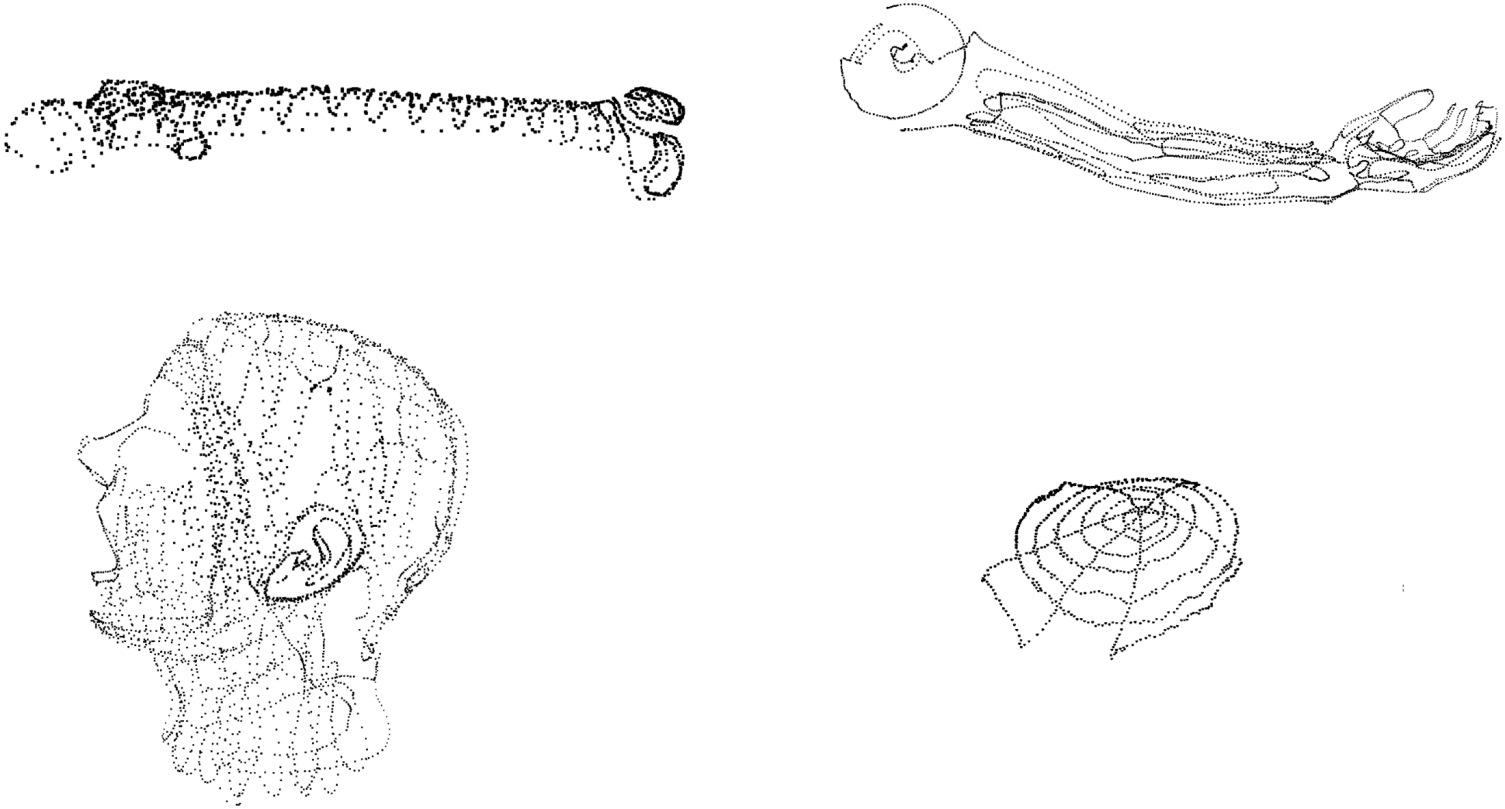
Point clouds from optical tracker in a 3D view.

The values of coordinates within the optical tracker system are collected by sliding a stylus tool against the phantom surface. Sample point cloud in a 3D view is shown in Fig 5. One can see the trajectories of the stylus tool recorded during the points collection.

**ICP registration** The image registration that matches the ToF-camera image (Fig 4) to the CT phantom image (Fig 3) and the point cloud acquired by the optical tracker (Fig 5) is based on the meshes geometry. For this, the ICP (*Iterative Closest Point*) technique has been chosen [53]. In our study the initial pose is defined by the camera set-up pre-alignment step. The structure of acquired datasets imposes a point-to-point solution [45, 54].

The ICP technique consists of six steps [54]: (1) selection of set of points to be registered in one or both meshes, (2) matching the points between meshes, (3) weighting the corresponding pairs of points, (4) rejecting certain pairs, (5) assigning an error metrics based on pairs of points, and (6) minimizing the error metrics. Each of these steps can differently affect the registration performance. In our approach various methods have been employed at these steps. The selection of points to be registered in both meshes is performed in the pre-segmentation step. As recommended in [43], all points yielded by the pre-segmentation are used for further registration. The matching of points between meshes is performed by a *k*-dimensional tree algorithm [55] applied in order to increase the speed of the nearest neighbour search. The constant weight used to describe the corresponding pairs is then followed by rejecting 5% of the worst pairs of points in terms of the Euclidean distance. The root-mean-square error (RMSE) is used to evaluate distances between corresponding points. The optimal rotation between points is found using the Singular Value Decomposition (SVD) [56]. Since we do not focus on the convergence speed of the ICP algorithm, the registration is preceded by the pre-alignment resulting in initial rotation matrix estimation.

## Results

The accuracy and robustness of the registration procedure in medical applications were tested using four phantoms introduced in Section Materials and Methods. In each case all three acquisition techniques (ToF-camera, optical tracker, CT) produced the point clouds. We compared and evaluated registration accuracies of each of three pairs of datasets in terms of Hausdorff distance [34] and mean absolute distance [57]. For given two finite point sets (surfaces) *A* = {**a**_1_, …,**a**_*n*_} and *B* = {**b**_1_, …,**b**_*m*_}, the directed Hausdorff distance (*HD*) is defined as:

$$\begin{array}{c} H\;D\left(A,B\right)=\max_{a_{i}\in A}\min_{b_{j}\in B}∥a_{i}-b_{j}∥, \end{array}$$ (6)

where ‖⋅‖ is the Euclidean norm on the points of *A* and *B*. The mean absolute distance (*MAD*) for a pair of surfaces *A* and *B* is the mean of the distance values from *A* to *B* for all *n* voxels in *A*:

$$\begin{array}{c} M\;AD\left(A,B\right)=\frac{1}{n}\sum_{i=1}^{n}\min_{b_{j}\in B}∥a_{i}-b_{j}∥. \end{array}$$ (7)

The pairwise registration between each: ToF, CT, and optical tracker in both directions yielded six pairs of metrics values labelled as: “CT to Opt”, “Opt to CT”, “Opt to ToF”, “ToF to Opt”, “ToF to CT”, and “CT to ToF”. Precision of the ToF-camera calibration step and its influence on the accuracy of further inter-sensor analysis and registration was evaluated, as described below.

Two system set-ups varying in position of the phantoms, ToF-camera and optical tracker are denoted as Pos.#1 and Pos.#2 (#1 and #2 in Fig 2, respectively) in further discussion and presentation of results. The numerical results for the manual correction influence analysis are labelled Raw (if no correction is introduced to the cloud of points) and Corrected (if manual corrections are introduced).

### Calibration accuracy

**Fig 6:**
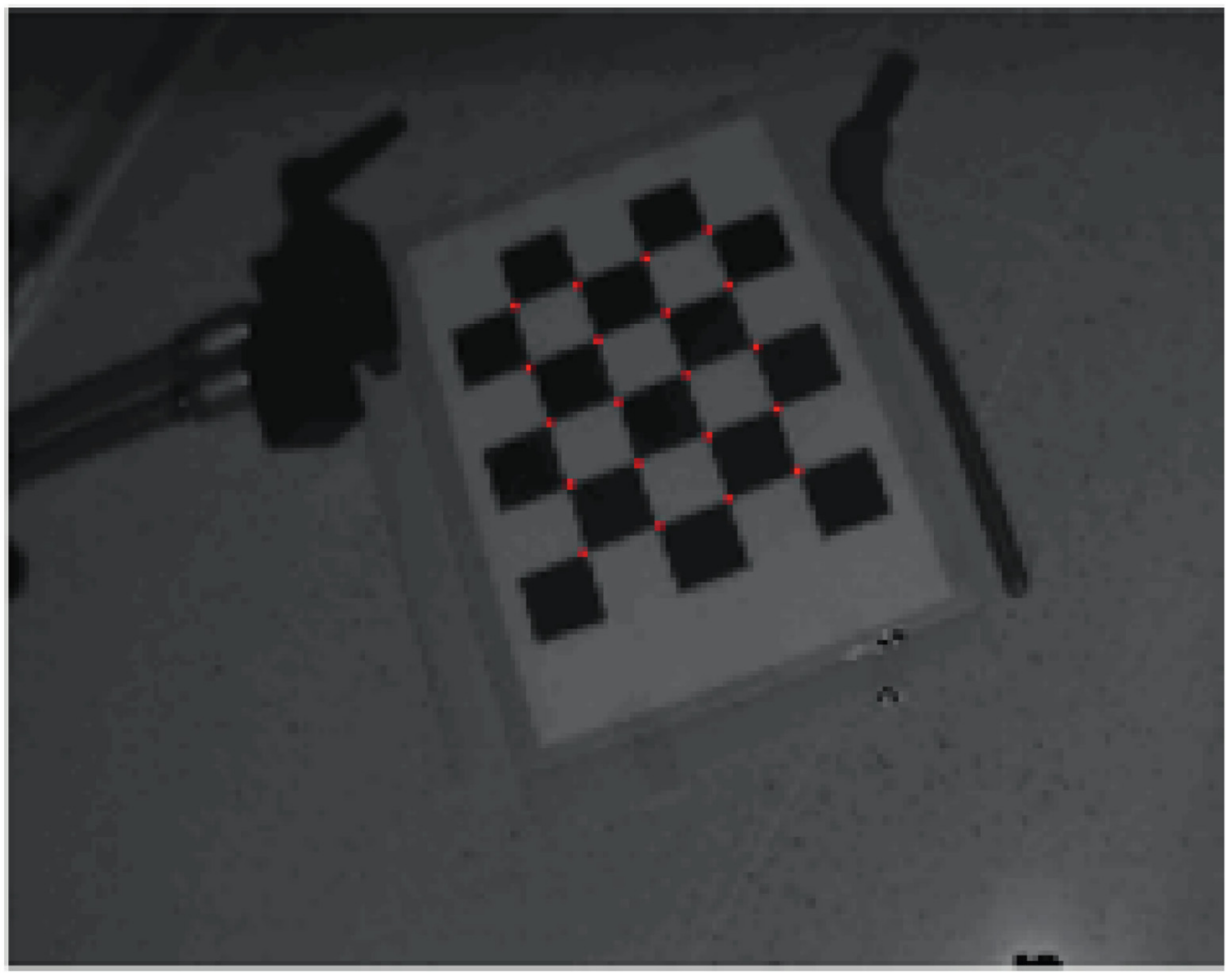
ToF-camera amplitude image with (rounded to full pixels) corresponding 3D points.

To evaluate the calibration stage, the intrinsic and extrinsic parameters were determined with respect to the reference coordinate system Z defined by the marker fixed at the camera located in position #1 (Fig 2). Then, the chessboard corners (testing points), whose positions were acquired by the stylus tool in the Z system, were projected (PnP, as described in Section Registration system) to the 2D amplitude image J (Fig 6):

$$\begin{array}{c} P_{i}^{J}=\text{PnP}\left(P_{i}^{Z},M_{I},M_{E}\right), \end{array}$$ (8)

where *M*_*I*_ and *M*_*E*_ are the matrices of intrinsics and extrinsics. Coordinates of both, 2D testing points found in the amplitude image and projections of their corresponding 3D points, were compared in terms of their Euclidean distance. The mean distance is shown in the first row of Table 1.

**Table 1:** Mean projection errors obtained using two camera positions for calibration and validation (Fig 2).

Calibration position	Validation position	Mean error ± std. dev. [px]
#1	#1	0.26±0.13
#1	#2	0.27±0.08
#2	#1	0.66±0.21
#2	#2	0.15±0.08

Then, the spatial relation was changed by relocating all the components: navigation tracker, ToF-camera and the chessboard. Coordinates of the testing chessboard points were transformed to the Z coordinate system using previous calibration parameters. At position #2 of the camera (Fig 2), projection of the image J was found as:

$$\begin{array}{c} P_{i}^{J}=\text{PnP}\left({\left(T_{G}^{Z}\right)}_{\tau }P_{i}^{G},M_{I},M_{E}\right), \end{array}$$ (9)

where *M*_*I*_ and *M*_*E*_ were obtained from camera position #1, and ${\left(T_{G}^{Z}\right)}_{\tau }$ denotes the current camera position (#2) in the global coordinate system (see Section Registration system). Again, Euclidean distances were determined between the 2D testing points and the projections of their corresponding 3D points, whose location was changed.

The entire procedure described above was repeated after switching positions #1 and #2. The projection errors obtained during all four validation steps are presented in Table 1. As expected, the calibration process is saddled with a low transformation error in the image plane, whereas the depth error is much higher. The obtained pre-alignment enables a further robust registration step. This analysis is specially important for symmetrical/pseudo-symmetrical structures where the ICP technique leads to an error resulting from rotation perpendicular to the axis of the visualized object. On the other hand, the depth error caused by the calibration step can easily be corrected by the ICP technique.

The obtained misalignments between ToF and optical tracker point clouds are presented in Table 2. The *HD* and *MAD* values are determined in three variants of set *A* from Eqs (6) and (7): with 0%, 3% and 10% (denoted as *p*_*m*_) of the worst matches rejected [13]. The relatively large misalignment results are improved by further surface registration step in terms of the ICP technique.

**Table 2:** ToF-to-optical tracker misalignment summary after ToF camera calibration.

	*HD* [mm]			*MAD* [mm]		
*p*_*m*_	10%	3%	0%	10%	3%	0%
Femur	15.8	26.9	48.8	7.0	8.0	8.9
Upper limb	47.6	58.9	68.2	15.6	18.2	19.6
Head	18.9	23.2	32.6	8.7	9.6	10.1
Breast	89.1	105.9	116.6	40.0	44.1	46.1

Results obtained for raw data with *p*_*m*_ equal to 10%, 3% and none of the worst matches removed.

### ICP registration results

**Fig 7:**
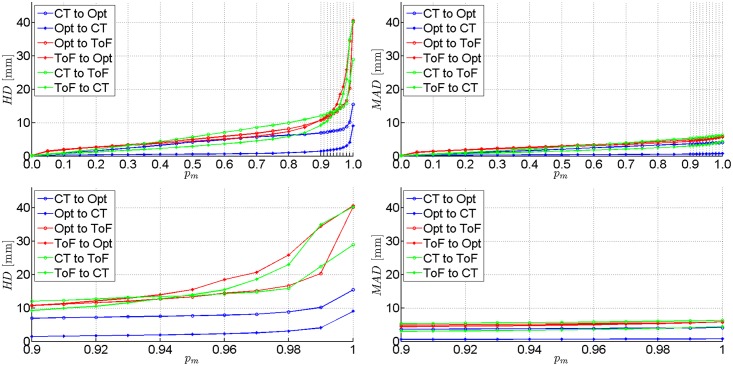
Results of the first experimental set-up on femoral phantom (Pos.#1). *HD* and *MAD* between each pair of registered point clouds with the percentile *p*_*m*_ of worst matches removed. Plotted at the full range (top) and zoomed to the [0.9, 1.0] range of *p*_*m*_ (bottom).

**Fig 8:**
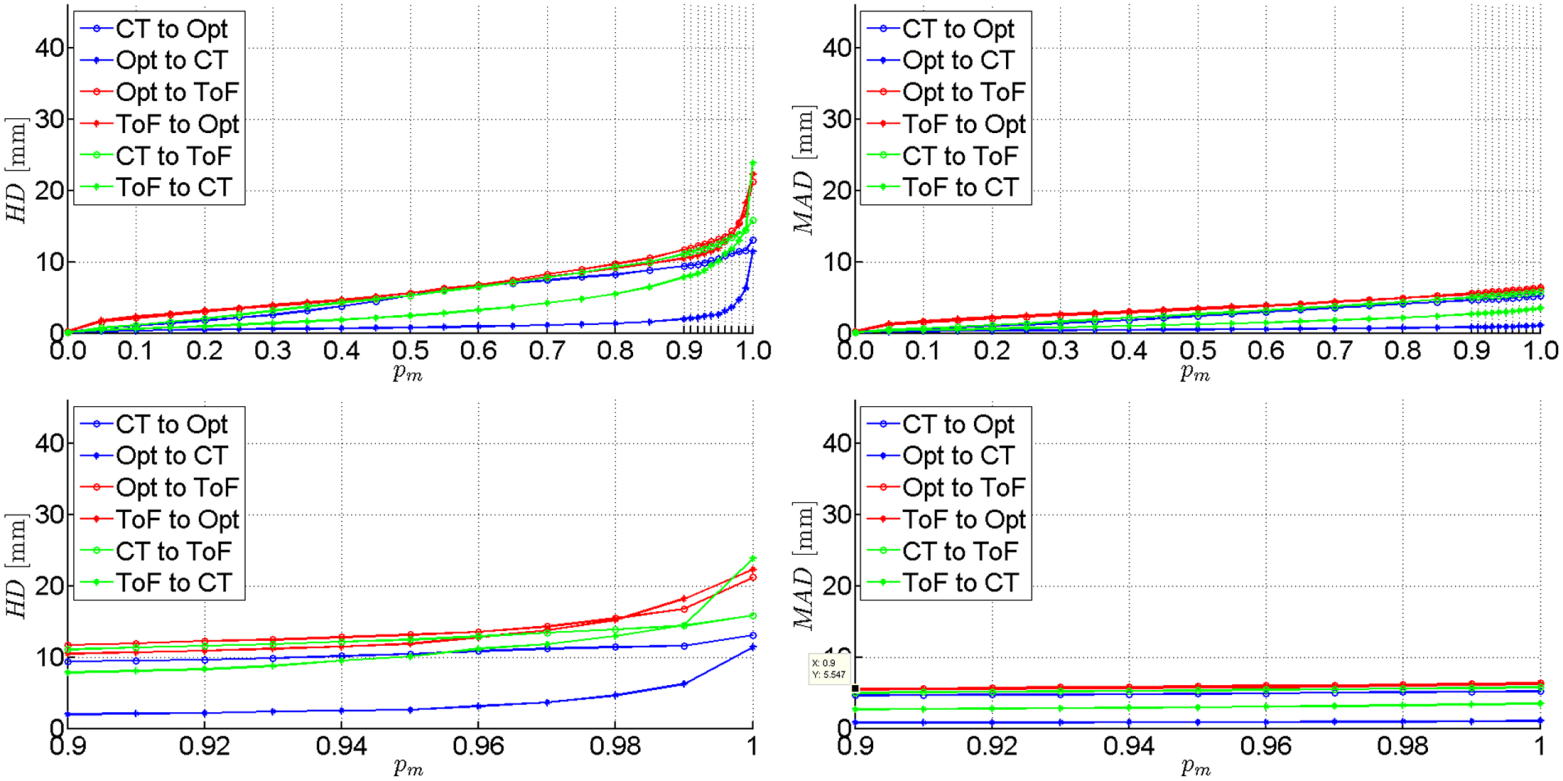
Results of the second experimental set-up on femoral phantom (Pos.#2). *HD* and *MAD* between each pair of registered point clouds with the percentile *p*_*m*_ of worst matches removed. Plotted at the full range (top) and zoomed to the [0.9, 1.0] range of *p*_*m*_ (bottom).

The ICP registration accuracies of each of the three pairs of data were evaluated for all phantoms. Results obtained for two experimental set-ups Pos.#1 and Pos.#2 are shown in Table 3. We present *HD* and *MAD* values calculated for registered point clouds if the worst 10%, 3% and 0% percentile of points (denoted as *p*_*m*_) is rejected. Extended *HD* and *MAD* results presentation for femoral phantom are provided in Figs 7 and 8 As the ICP registration is not symmetrical, the *HD* and *MAD* values are computed twice for each pair of data (in both directions).

**Table 3:** ICP registration results in various ToF camera positions.

	Pos.#1						Pos.#2					
	*HD* [mm]			*MAD* [mm]			*HD* [mm]			*MAD* [mm]		
*p*_*m*_	10%	3%	0%	10%	3%	0%	10%	3%	0%	10%	3%	0%
Femur												
CT to Opt	6.9	8.2	15.4	3.6	3.9	4.1	9.4	11.2	13.1	4.6	5.0	5.2
Opt to CT	**1.4**	**2.4**	**8.6**	**0.6**	**0.7**	**0.8**	**2.0**	**3.6**	**11.4**	**0.8**	**0.9**	**1.1**
Opt to ToF	13.1	20.1	45.1	4.8	5.4	5.9	11.7	14.3	21.2	5.5	6.1	6.4
ToF to Opt	10.8	20.6	40.6	4.4	5.1	5.9	10.5	13.8	22.3	5.4	5.9	6.2
CT to ToF	12.0	14.8	28.9	5.3	5.9	6.3	11.1	13.4	15.8	5.0	5.5	5.8
ToF to CT	9.2	18.6	40.2	3.0	3.7	4.5	7.8	11.8	23.8	2.7	3.2	3.5
Upper limb												
CT to Opt	4.9	6.5	7.8	2.4	2.6	2.8	4.9	6.5	7.8	2.4	2.6	2.8
Opt to CT	**1.7**	**2.9**	**6.7**	**0.7**	**0.8**	**0.9**	**1.7**	**2.9**	**6.7**	**0.7**	**0.8**	**0.9**
Opt to ToF	10.4	19.4	32.1	4.1	4.8	5.4	16.3	24.9	40.1	5.3	6.3	7.1
ToF to Opt	10.6	19.2	33.0	4.2	4.9	5.5	18.0	27.3	41.9	5.7	6.9	7.7
CT to ToF	19.1	28.4	43.4	6.9	8.1	8.8	22.1	29.1	41.4	8.8	9.9	10.6
ToF to CT	21.3	28.8	46.0	6.8	8.1	8.8	23.9	31.1	44.0	8.9	10.2	10.9
Head												
CT to Opt	5.5	6.1	8.4	3.2	3.4	3.5	5.5	6.1	8.4	3.2	3.4	3.5
Opt to CT	**1.1**	**2.2**	**6.5**	**0.6**	**0.6**	**0.7**	**1.1**	**2.2**	**6.5**	**0.6**	**0.6**	**0.7**
Opt to ToF	11.5	19.8	28.2	3.6	4.4	5.0	8.3	14.7	36.4	2.7	3.3	3.8
ToF to Opt	8.5	13.8	24.6	3.5	4.0	4.4	9.8	19.4	37.0	3.0	3.8	4.4
CT to ToF	11.6	13.2	18.4	7.1	7.5	7.7	10.8	16.2	73.2	5.8	6.3	7.0
ToF to CT	3.4	5.1	8.6	1.3	1.5	1.6	5.2	18.7	75.0	1.8	2.2	3.2
Breast												
CT to Opt	15.9	19.5	26.2	7.1	7.9	8.3	15.9	19.5	26.2	7.1	7.9	8.3
Opt to CT	**1.7**	**2.5**	**5.3**	**0.7**	**0.8**	**0.9**	**1.7**	**2.5**	**5.3**	**0.7**	**0.8**	**0.9**
Opt to ToF	9.8	24.9	50.5	4.6	5.2	6.2	11.6	22.1	39.8	5.1	5.9	6.5
ToF to Opt	16.3	32.5	50.8	6.8	8.0	8.9	18.3	28.5	37.7	7.1	8.2	8.9
CT to ToF	24.8	38.3	52.8	8.9	10.5	11.5	26.6	38.8	55.1	10.0	11.5	12.5
ToF to CT	32.0	45.8	58.8	8.7	10.8	12.0	34.1	46.0	60.0	10.8	12.8	14.0

Results are estimated for all ICP registrations performed over raw data with 10%, 3% and 0% of the worst matches (*p*_*m*_) removed. Pos.#1 and Pos.#2 refer to two different positions of the phantom and sensors.

Although it is common to reject the worst 10% of matches to get accurate results [13], one can see in Table 3 that only last 3% of matches have the greatest impact on the *HD* value.

The best alignment results at *p*_*m*_ = 10% are obtained if registration from the tracker to CT is performed, yielding *HD* values equal to 1.1 mm and 2.4 mm in the best and the worst case, respectively. In the opposite direction the *HD* results are substantially higher (2.4–15.9 mm). However, if the ToF-sensor data are registered, the *HD* values range from 3.4 to 34.1 mm.

The *MAD* metrics indicates the level of shape correspondence between surfaces yielded by each acquisition technique. The *MAD* consistently below 1 mm in case of a CT and optical tracker registration ought to be considered as rewarding. In general, most of the mean absolute distances are below 1 cm. The only exceptions from the above rule are related to the ToF camera and they result from its acquisition inaccuracy [21].

**Fig 9:**
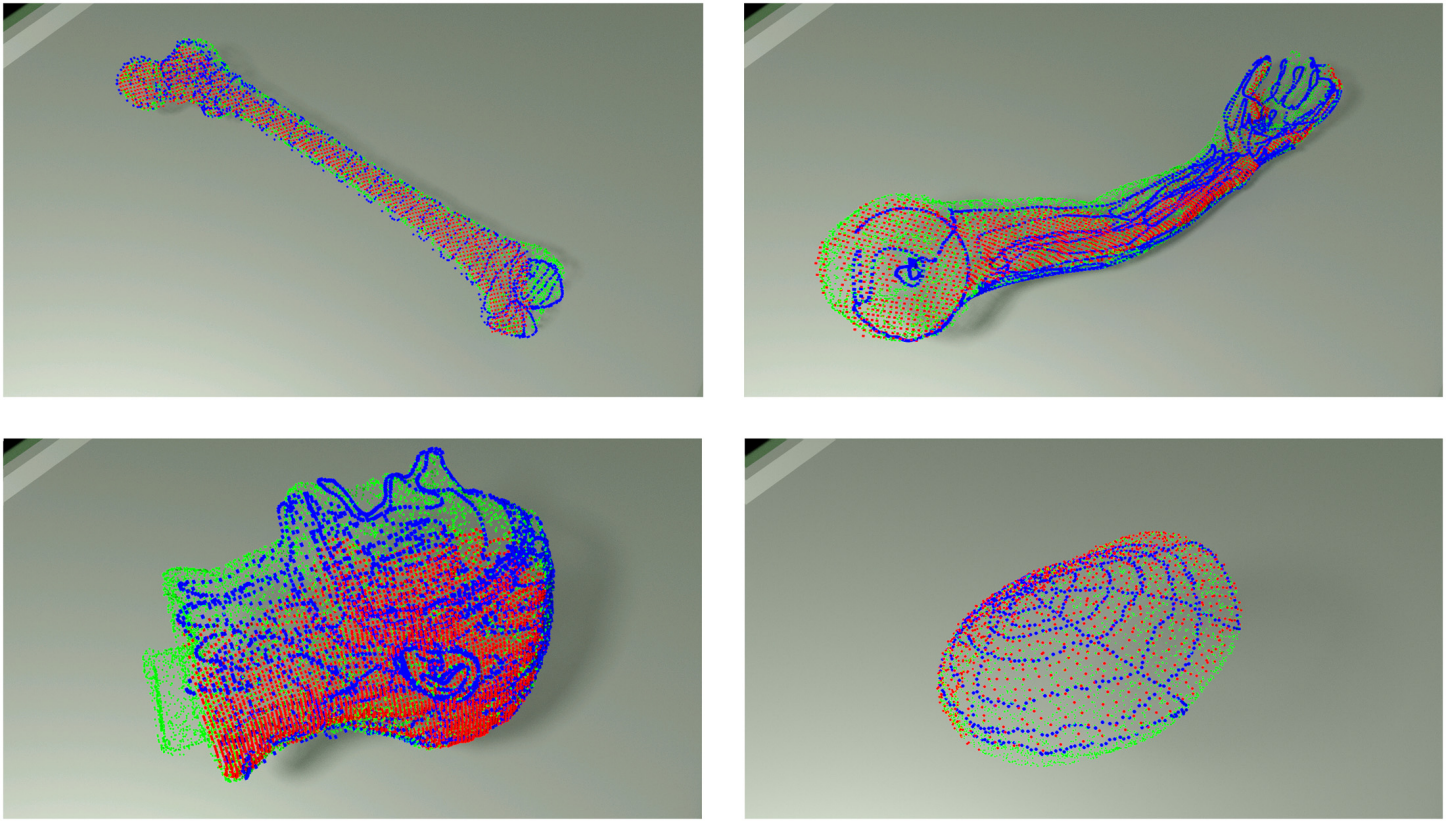
Visualisation of the registration results. ToF data (red), CT data (green), optical tracker data (blue).

The visualisations of all three registered point clouds are shown for each phantom in Fig 9.

### The influence of manual data correction

**Fig 10:**
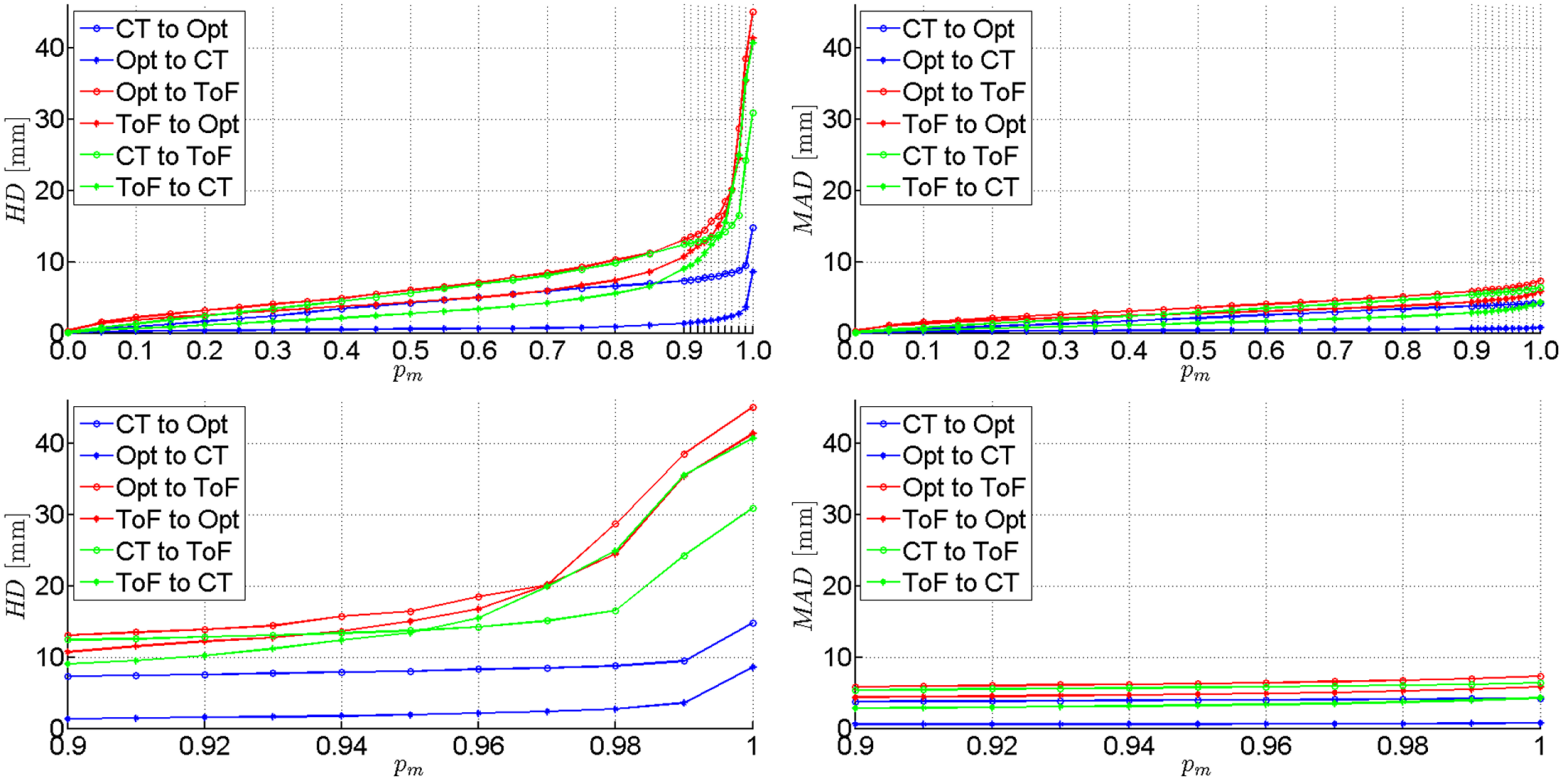
Results of the first experimental set-up on femoral phantom with manually removed outliers (Pos.#1, Corrected). *HD* and *MAD* between each pair of registered point clouds with the percentile *p*_*m*_ of worst matches removed. Plotted at the full range (top) and zoomed to the [0.9, 1.0] range of *p*_*m*_ (bottom).

Since in medical applications automated procedures are often followed by manual corrections [58], we included the adequate analysis in our experiments. Two registered point clouds: ToF-data and optical tracker data were pre-processed by the manual removal of outliers. The points were removed by a medical and computer vision expert. The obtained results are labelled as Corrected in Table 4, as well as are shown in Fig 10. No significant improvement of the results is observed. The *HD* at *p*_*m*_ = 10% after manual correction differs from the raw data results by not more than 1.8 mm, yet in most cases the difference barely exceeds 0.5 mm. Taking *MAD* into account, the influence of manual data correction is even less noticeable—respective values differs mostly not more than by 0.2 mm.

**Table 4:** The influence of manual data corrections on ICP registration.

	Raw						Corrected					
	*HD* [mm]			*MAD* [mm]			*HD* [mm]			*MAD* [mm]		
*p*_*m*_	10%	3%	0%	10%	3%	0%	10%	3%	0%	10%	3%	0%
Femur												
CT to Opt	6.9	8.2	15.4	3.6	3.9	4.1	7.3	8.5	14.8	3.8	4.1	4.2
Opt to CT	**1.4**	**2.4**	**8.6**	**0.6**	**0.7**	**0.8**	**1.4**	**2.4**	**8.6**	**0.6**	**0.7**	**0.8**
Opt to ToF	13.1	20.1	45.1	4.8	5.4	5.9	13.1	20.1	45.1	5.9	6.6	7.4
ToF to Opt	10.8	20.6	40.6	4.4	5.1	5.9	10.7	20.2	41.3	4.4	5.1	5.8
CT to ToF	12.0	14.8	28.9	5.3	5.9	6.3	12.4	15.1	30.9	5.4	5.9	6.4
ToF to CT	9.2	18.6	40.2	3.0	3.7	4.5	9.0	19.9	40.7	2.8	3.5	4.3
Upper limb												
CT to Opt	4.9	6.5	7.8	2.4	2.6	2.8	5.0	5.9	8.0	2.5	2.7	2.8
Opt to CT	**1.7**	**2.9**	**6.7**	**0.7**	**0.8**	**0.9**	**1.7**	**2.9**	**6.6**	**0.7**	**0.8**	**0.9**
Opt to ToF	10.4	19.4	32.1	4.1	4.8	5.4	9.9	19.6	32.8	4.0	4.7	5.3
ToF to Opt	10.6	19.2	33.0	4.2	4.9	5.5	10.0	19.5	32.5	4.0	4.7	5.3
CT to ToF	19.1	28.4	43.4	6.9	8.1	8.8	19.1	28.3	43.4	6.9	8.1	8.8
ToF to CT	21.3	28.8	46.0	6.8	8.1	8.8	21.4	28.9	45.9	6.8	8.1	8.8
Head												
CT to Opt	5.5	6.1	8.4	3.2	3.4	3.5	5.5	6.0	8.4	3.2	3.4	3.5
Opt to CT	**1.1**	**2.2**	**6.5**	**0.6**	**0.6**	**0.7**	**2.4**	**3.4**	**7.3**	**1.1**	**1.2**	**1.3**
Opt to ToF	11.5	19.8	28.2	3.6	4.4	5.0	11.5	20.0	28.7	3.6	4.5	5.0
ToF to Opt	8.5	13.8	24.6	3.5	4.0	4.4	8.6	13.8	24.4	3.5	4.0	4.4
CT to ToF	11.6	13.2	18.4	7.1	7.5	7.7	11.6	13.2	18.4	7.1	7.5	7.7
ToF to CT	3.4	5.1	8.6	1.3	1.5	1.6	3.4	5.1	8.6	1.3	1.5	1.6
Breast												
CT to Opt	15.9	19.5	26.2	7.1	7.9	8.3	14.1	18.0	23.1	6.4	7.1	7.4
Opt to CT	**1.7**	**2.5**	**5.3**	**0.7**	**0.8**	**0.9**	**1.6**	**2.3**	**5.2**	**0.7**	**0.8**	**0.8**
Opt to ToF	9.8	24.9	50.5	4.6	5.2	6.2	9.5	26.3	50.4	4.5	5.2	6.1
ToF to Opt	16.3	32.5	50.8	6.8	8.0	8.9	15.9	31.8	50.2	6.6	7.8	8.7
CT to ToF	24.8	38.3	52.8	8.9	10.5	11.5	24.8	38.3	52.7	8.9	10.5	11.5
ToF to CT	32.0	45.8	58.8	8.7	10.8	12.0	32.0	45.8	58.9	8.7	10.8	12.0

Results are estimated for all ICP registrations performed over raw data (Raw) and data with outliers removed manually (Corrected) with 10%, 3% and 0% of the worst matches (*p*_*m*_) removed. All experiments for the ToF camera position Pos.#1.

## Discussion

The goal of this paper was to investigate the surface matching using CT, ToF and the optical navigation system in a real environment and to test its applicability for medical data registration. The study results provide a comparison of accuracy estimates for three combinations of surface alignment. Since the phantoms were scanned repeatedly in various positions, we got also some information on the robustness of the registration systems. Finally, the impact of manual correction of the image data on the overall accuracy was tested. Although all the analysed image modalities and sensor data are already applied in medical field [47, 48], the introduced combination of them stands for the original contribution and features some significant potential for image guided surgery systems.

For the numerical analysis we used the ICP algorithm enabling fast and robust rigid registration whose accuracies evaluated by Hausdorff and *MAD* distances proved usefulness of the multi-sensors visualization system. However, it has to be noticed, that in order to achieve high quality of the multi-sensors system, additional issues have to be addressed.

Since the overall idea of the study was to determine the maximum accuracy of the multi-sensor system, the results originate from the phantom experiments. However, in clinical settings one deals with various types of patient-related motions, time constrains, unforeseen events that may challenge the workflow. Further studies are required to improve the accuracy by employing a second ToF-camera [48].

Shapes of the phantoms are non-symmetrical and variform, yet the phantoms’ materials are very easy to segment from CT scans itself. Diverse shapes resemble the limitations of measurement performed with an optical tracker, indicating the angle of optical marker, number of measurement points, or the difficulties of the object surface scanning by the optical marker.

The ToF-sensor is a relatively cheap and safe tool to align the intra-operative surface onto the pre-segmented CT data and can be used in cooperation with the optical tracker or as a stand-alone device. However, to employ it in medical application some of its constraints have to be considered. According to our registration accuracy measurements, despite the relatively low resolution of the camera’s CCD and high level of noise, the ToF-sensor is suitable mostly for the pre-alignment of registered surfaces. The ToF sensor calibration is required in order to obtain direct transformation formula between both acquisition systems (ToF-sensor and optical tracker). It is particularly important for the analysis of the almost symmetrical structures for which the ICP algorithm may result in a reversal along their axis of symmetry.

The effect of depth inhomogeneity leading to wrong distance measurements at object boundaries has to be reduced in order to obtain the accurate ToF point cloud segmentation. It can be done on the basis of a confidence map provided by the sensor. This confidence information is also important in the context of curvature of visible space at the depth image edges. The accuracy of a ToF-camera varies with respect to the direction of the recorded structure. The depth error is substantially higher than the error in the image plane [21]. Thus, the spatial orientation of segmented structure is relatively correctly measured, whilst the translation in depth-direction causes most of the total error.

It is also noteworthy, that if the optical tracker and the ToF-camera using infra-red light are running together at the same time, they may disturb each other’s measurements. The interferences may occur if the optical marker is located exactly between the lenses of the devices. The exact analysis of the interferences will be performed in future studies.

Calibrated ToF-camera is a device which could be potentially deployed at the stage of positioning an object in the navigation system space. The results from Tables 3 and 4 confirm, that the worst alignment between optical tracker and ToF camera (1.8 cm, *p*_*m*_ = 10%) is at the level of acceptance even in some medical fields [11]. Moreover, our experiments showed that if the number of rejected points is decreased to 3%, the misalignment is still acceptable. A period of surface acquisition is much shorter than the duration of pointing the stylus tool at the body’s landmarks. Also, the point cloud obtained by ToF-camera contains significantly more elements than the usual number of landmarks. The registration process performed by surface matching yields results not worse than landmark-based registration [25]. This could allow the stylus tool to be replaced by a ToF-camera during object calibration. This will be investigated in the further research.

## Conclusion

In the paper, we present the first study on the abilities of three-modal surface registration using: CT scanning in a pre-operative mode, the SwissRanger SR4000 ToF-camera and the Polaris Spectra navigation system. The multi-sensors data analysis in medical field opens new possibilities in minimally invasive surgery, giving the feedback concerning pre- and intra-operative data correspondence. The proposed experimental set-up gives the reader information concerning some limitations of the system, possible problems to be addressed and solutions how to deal with them. The obtained pairwise inter-modality alignment accuracies at a few millimeter range allow us to conclude, that together with technological advancement of ToF-cameras, they are going to be used increasingly in various fields of medicine.
